# 41224 REDUCED FRONTOSTRIATAL FUNCTIONAL CONNECTIVITY IN 41- TO 70-YEAR-OLD ADULTS WITH HIV

**DOI:** 10.1017/cts.2021.436

**Published:** 2021-03-30

**Authors:** Shiva Hassanzadeh-Behbahani, Fan Nils Yang, Margarita Bronshteyn, Matthew Dawson, Princy Kumar, John VanMeter, David J. Moore, Ronald J. Ellis, Xiong Jiang

**Affiliations:** 1 Georgetown University Medical Center; 2 University of California San Diego

## Abstract

ABSTRACT IMPACT: The knowledge acquired from my research can inform the development of early diagnostic methods for HIV-associated neurocognitive disorders. OBJECTIVES/GOALS: In the era of combination antiretroviral therapy (cART), the prevalence of HIV-associated neurocognitive disorders (HAND) remains high but the neural mechanisms are unclear. We examined whether older people with HIV (PWH) with minimal cognitive impairment have reduced functional connectivity in frontostriatal circuits compared to controls. METHODS/STUDY POPULATION: 99 PWH (mean age 56.6 years, 75% male, 62% Black, mean duration of HIV-infection 26.2 years ±9.3, 90% viral load <50 copies, 98% on stable cART) and 38 demographically-comparable controls (mean age 54.5 years, 71% male, 58% Black) participated in a cross-sectional study. A 7-domain neuropsychological battery and an Activities of Daily Living index were used to determine HAND diagnoses: 32 PWH met criteria for asymptomatic to mild HAND. Motor skill was assessed using the Grooved Pegboard Test by measuring performance speed. Structural MRI and resting-state functional MRI were collected. Seed-to-voxel analyses were conducted using 4 distinct regions in the striatum as seed regions. We used a voxel threshold of p<0.001 and cluster threshold of p<0.05 (FDR-corrected) after controlling for demographic variables. RESULTS/ANTICIPATED RESULTS: Compared to controls, PWH had lower resting state functional connectivity between the default mode region of the striatum (i.e., medial caudate) and bilateral superior frontal gyrus, supplementary motor cortex and paracingulate gyrus (p<0.05; cluster size: 567 voxels). Also, compared to controls, PWH had reduced resting state functional connectivity between the motor division of the striatum (i.e., posterior putamen) and anterior cingulate cortex and left supplementary motor cortex (p<0.05, cluster size: 405 voxels). Performance speed on the Grooved Pegboard motor test negatively correlated with functional connectivity between the motor region of the striatum and supplementary motor frontal regions in all participants (Spearman’s rho=-0.18, p=0.04). DISCUSSION/SIGNIFICANCE OF FINDINGS: Our results support the hypothesis that frontostriatal abnormalities are widely present in PWH and might play a key role in HAND development. Our data suggest that dysfunction within the frontostriatal circuits may be involved in motor impairment in PWH, and ongoing inflammation may contribute to motor impairment and frontostriatal injury.

